# An Indigenous critique: Expanding sociology and recognizing unique Indigenous knowledge

**DOI:** 10.3389/fsoc.2022.1047812

**Published:** 2022-11-29

**Authors:** Ried E Mackay

**Affiliations:** Department of Sociology, Texas A&M University, College Station, TX, United States

**Keywords:** race and ethnicity, Indigenous theory, postcolonial, Native Americans, systemic racism, multi-raciality, Global South, Global North bias

## Abstract

**Introduction:**

This essay suggests that sociologists should integrate into their critical research work on the Americas an Indigenous critique/method based on Indigenous knowledge. As a mixed Indigenous scholar, I have been frustrated by the lack of frameworks based explicitly on Indigenous knowledge rather than merely referencing that knowledge.

**Methods:**

Strong foundations of ancient Indigenous thought and philosophical tradition—which often differs dramatically from Western traditions—are identified and explored through three concepts: Ch'ixi, the Indigenous pragmatic, and Mexica concepts of Truth. These are identified and discussed using authoritative historical and contemporary sources. I provide potential pathways for usage of these concepts in the results and discussion. Arguments and controversy for accepting the validity of Indigenous sources are also addressed.

**Results and discussion:**

Discussions of specific empirical questions and puzzles related to already familiar concepts and analyses such as systemic racism theory, multi-raciality, religion, and postcolonial theory are explored. The paper concludes that Indigenous theory is underexplored but is critical to liberation of Indigenous people and has legitimate academic value that scholars need to recognize.

## Introduction

*Is there, perchance, any truth to our words here?*

I begin this paper as the ancient Tlamatinime (“someone who knows something;” philosophers) of the Mexica (Aztec) civilization began their own contemplations (Léon-Portilla, 1963: 70, 99, 112, 132). The great thinkers of the pre-invasion Indigenous Americans would begin their scholarly pursuits by first expressing a philosophical doubt as to whether any of what followed could be considered the *truth*. The *truth* to Indigenous Americans—of what we now call the North and South Americas—is starkly different from that of traditional Euro-Western thought, it does not rely on hard dichotomies and absolutes. This ongoing focus on Euro-Western thought obscures that the population of these two continents was estimated to be at least 60.5 million and potentially as high as 78 million, equivalent to estimates in Europe at the time, and the subsequent depopulation from disease and violence caused measurable global climate impacts (Koch et al., 2019). Despite this population size and sophistication of intellectual traditions, the focus decidedly remains on European thought and tradition. Recognizing and incorporating the traditions of the Americas is incredibly important to more fully understand the Indigenous American lived experience and lead the academies of this hemisphere into a decolonial future (Sánchez-Antonio, 2022).

Over a year ago as I, a mixed Indigenous person, sat in the shade of towering and impressive monuments of the Ancestors and Gods—during another sickness ravaging Indigenous people and many others—I began to think about how Global North sociology can begin to truly integrate Indigenous peoples and their knowledge into the discipline. Indigenous scholars inside and out of sociology, and their non-Indigenous allies, are doing important work to bring recognition and liberation to Indigenous peoples, however Indigenous knowledge systems are still not allowed to stand on their own merits or explored in their own unique frameworks to reveal and answer new, and old, and questions. Rather, they are relegated to a secondary status within discussions and analyses. Recognizing Indigenous knowledge isn't simply about recognizing the knowledge as valid, it is about fully integrating that knowledge and investigating sociological, biological, theological and other processes from a specifically Indigenous point of view using Indigenous frameworks. This is important for the liberation of Indigenous peoples, if Indigenous knowledge and philosophies are always relegated to a secondary status or only used by a few specialists, then Indigenous people will continue to be assumed to be savage, primitive, and without valuable contribution to the scholarly community thereby perpetuating the deeply entrenched white racial framing of Indigenous people and culture (Mackay and Feagin, 2022).

This call for recognition of Indigenous philosophies and frameworks is already an established tradition, but it has received little attention by much of Global North scholarship. Scholars like Jairo Fúnez-Flores are publishing important articles that highlight the strong and determined resistance of Global North academies and institutions to Indigenous knowledge and frameworks (Fúnez-Flores, 2022) as well as engaging with these ideas in a public sociology through the medium of social networks like Twitter. Additionally, the important canon of work from prominent scholar Pablo González Casanova—father of sociology in Mexico and Zapatista comandante—constitute what he calls the Theory of the Jungle/Theory of the Rain Forest (González Casanova, 1998). This Rain Forest Theory encapsulates different Indigenous philosophies and elements of Etuaptmumk—two-eyed seeing (Kutz and Tomaselli, 2019)—and elevates them to a legitimate status equal to that of the Western traditions. The theme of his long career and allyship with Indigenous peoples and movements demands that we recognize the knowledge and philosophies of Indigenous peoples as theoretical contributions themselves, not merely in their political and ideological forms (Oropeza, 2022).

The Zapatistas have made this a core tenant of their movement by incorporating Indigenous philosophies and matriarchal leadership into their struggle against colonialism and oppression. The Zapatista movement is not only resurrecting these Indigenous philosophies—they are applying them on the ground to free Indigenous Americans (North and South) from the oppressions of ongoing colonialism (Schools for Chiapas, 2022; Sánchez-Antonio, 2022). Though epistemic justice (de Sousa Santos, 2014) is critical, this does not mean that European and US knowledge is disavowed, ignored, or otherwise repressed—that is a Westernized essentialist notion (Fúnez-Flores, 2022:5–7) which is in direct opposition to the plurality of thought and being that Indigenous traditions advocate for as an inherent principle to life and society (Pratt, 2002; Sánchez-Antonio, 2022).

As this essay is a recognition of oppressed and marginalized knowledge and thought it centers the long-ignored epistemologies of Indigenous communities and is a small contribution toward liberating Indigenous knowledges from ongoing epistemicide and culturicide (Fenelon, 2014; de Sousa Santos, 2014; Huaman and Brayboy, 2017; Leung and López-McKnight, 2020; Sánchez-Antonio, 2022). It is one addition to the larger body of knowledge that must be developed to resurrect and implement Indigenous millennia-knowledge (Sánchez-Antonio, 2022), through the contributions of the community this will be possible. Individual contributions in service to the larger community is a core aspect of the Global South tradition of comunalidad (Barkin, 2022). This paper recognizes, and refutes, the long-held elevation of Euro-Western knowledge and frameworks above that of Indigenous peoples and knowledge, which has occurred through an explicitly racialized lens and denunciation of Indigenous knowledge as “savage” (Williams, 2012; Mignolo and Walsh, 2018; Feagin, 2020; Mackay and Feagin, 2022; Sánchez-Antonio, 2022).

My essay begins by identifying the strong foundations of Indigenous thought that Indigenous and Global South scholars are exploring. The millennia-knowledge presented here was created in various social, historical, and geographical contexts, and it continues to live and evolve. While Indigenous cultures vary throughout the Americas, there is a common thread of shared thought/philosophy that has been identified by scholars, like the prominent scholar VF Cordova—one of the first Indigenous American women to receive a PhD in philosophy in the US–and the Zapotec philosopher Juan Carlos Sánchez-Antonio. I then move into a discussion of the Indigenous pragmatic, whose millennia old foundations are identified by Cordova (2007), Pratt (2002), and Sánchez-Antonio (2022). These three scholars base their well-reasoned arguments on multiple sources and experiences that are extensively detailed in their socio-philosophical works. The essay then identifies and discusses the academic resistance to Indigenous thought that pervades the academic establishments of the Global North. I conclude with a discussion on how Indigenous frameworks and philosophies can be used on their own merits in sociological analyses and provide recommendations of potential future research pathways.

## Foundations of Indigenous thought

Indigenous knowledge has intentionally been racialized, obfuscated, and destroyed by invading colonial armies and communities for centuries (Tedlock, 1996; Cordova, 2007; Townsend, 2019). It has also been deliberately obscured by claims of Global North “firsts”—for example important aspects of contemporary philosophy, public health, medicine, and engineering are Indigenous inventions: American pragmatism, syringes, mouthwashes, rubber, cable suspension bridges, hammocks, raised-bed agriculture, snow goggles, and more (Pratt, 2002; Kiger, 2019; Roberts, 2020). While Indigenous communities throughout what is now called North and South America are beautifully unique and varied, various scholars in Indigenous philosophy, history, and archaeology have recognized that Indigenous thought frameworks and knowledge were shared, with regional variations by large cultural, political, and economic groupings and alliances—such as the ancient Mississippians, Calusa, Mexica, Maya, and Inca. These scholars identify shared social and philosophical aspects—architectural and philosophical similarities, shared agriculture traditions, shared linguistic evidence (e.g., trading languages, similar definitions of words, similar relations of words to histories and proverbs, etc.), and shared applications of mathematics and geometries. They argue that this indicates a shared and generalizable Indigenous thought structure, or at least an extensive collaboration of knowledge building between the two continents (Cordova, 2007; Dunbar-Ortiz, 2021:231; Graeber and Wengrow, 2021:141–148).

It is important to recognize that when discussing Indigenous philosophy, we cannot make the “mistake to apply the criteria of the cult of the individual, which prevails in modern Western European culture, to the more socialized efforts of the philosophers of other times and places” (Léon-Portilla, 1963:22–23). Understanding that these works of thought were likely a culmination of many thinkers in a community and school of thought over generations, rather than attributable to a singular person is important. This ethic of shared social and intellectual constructions is representative in the overall philosophy of communalidad that scholars (Barkin, 2022; Sánchez-Antonio, 2022) identify as important aspects of Indigenous being, in the past and particularly in the present.

Prominent scholars (Léon-Portilla, 1963:9; Leeming, 2013; Townsend, 2019:2–13; Graeber and Wengrow, 2021:49–55) also argue that the ideas presented in the works are legitimately Indigenous since the Euro-invaders had no reason to lie about these ideas in their quest to understand them—so that they could ultimately attempt to destroy those ideas. However, in an interesting twist, some scholars of religion have recently argued that those destructive efforts largely backfired and instead affirmed the Indigenous communities' already established views of the world, particularly through the commonly shared Indigenous concept of pluralism (Pratt, 2002; Cordova, 2007; Salomon and Urioste, 2010:1–13; Leeming, 2013).

## Indigenous philosophy, truth, and ch'ixi

Indigenous philosophy is grounded in contemplating an everchanging, interconnected, omnipresent, but ultimately unknowable universe and *truth* (Léon-Portilla, 1963; Maffie, 2014; Waters, 2015:11–17; Purcell, 2018:11–21). This truth is not a simple duality as is familiar in Euro-Western thought. Indigenous truth operates from recognition and integration of included thirds (Léon-Portilla, 1963; Maffie, 2014; Purcell, 2018; Rivera Cusicanqui, 2020): two things can form a third and/or one thing can inherently, necessarily, and peacefully consist of multiple equal parts. However, we do not have the vocabulary in English to fully understand the concept as Indigenous Aymara/Bolivian sociologist Silvia Rivera Cusicanqui (2020) illustrates:

…Hybridity assumes the possibility that from the mixture of two different beings a third completely new one can emerge…but the mule is a hybrid which cannot reproduce…*ch'ixi* on the contrary…expresses the parallel coexistence of multiple cultural differences that do not extinguish but instead antagonize and complement each other.

Ch'ixi demands that we shrug off the oppressive dualities of Western thought and accept that a reality exists where mutable, evolving, and ultimately unknowable “thirds” exist. Anzaldua (2012) highlights this idea of an included third in her seminal work *Borderlands* where she discusses mixing cultures and identity to create new ones. However, even here the idea of ch'ixi could complicate this work, something I will explore later in this essay.

The idea that truth and reality (i.e., life) are unknowable and always changing is beautifully illustrated in Mexica philosophy as “only do we awake to dream” (Léon-Portilla, 1963:71; Maffie, 2014:13, 27, 39–47, 51). The Tlamatinime do not imply we live in a dream world, only that we cannot understand all parts of reality; that we can only see certain aspects and therefore live a life of only seeing the parts rather than the whole as it truly exists. This acceptance that we necessarily cannot understand, discover, or conquer everything in our reality stands in contrast to the Euro-Western conceptualization that reality is entirely knowable, discoverable, and conquerable. The Indigenous approach does not insinuate our lives and realities are any less important; it doesn't suggest that our actions have no consequences or imply that we are somehow wrong in our knowledge of the world (Purcell, 2018:11–21), it recognizes that we cannot, and likely should not, know it all.

## Indigenous pragmatism encounters western imperialism

Scott L. Pratt argues that the Indigenous philosophical tradition has survived in the Global North and directly created the American philosophical tradition of pragmatism—he terms it “Native” pragmatism, but I prefer Indigenous as it encompasses both North and South American traditions. Pratt identifies four dimensions of Indigenous pragmatism and demonstrates how they have been used by Indigenous leaders over the centuries. Though he is a philosopher by training, his book includes multiple important sections on the racism and other socio-political contexts of colonialism that Indigenous people, and the Indigenous pragmatic, were and are encountering. Pratt's concept of the Indigenous pragmatic and its dimensions is supported by the lifelong work of Cordova and the work of Zapotec scholar Sánchez-Antonio. All three scholars separately argue that Indigenous concepts constitute full-fledged philosophy that can in turn be used for analyses of society, ethics, and more. Pratt's Indigenous pragmatism involves four key dimensions (Pratt, 2002:20–38; see Table 1): Interaction, Pluralism, Community, and Growth. While defined differently, Sánchez-Antonio discusses similar dimensions posited by the scholar Martínez Luna (2015).

**Table 1:** Dimensions of Indigenous pragmatism.

**Indigenous pragmatism**	
Interaction (geographical)	All organisms, objects, and beings interact with one another and are constituted by and continuous with those interactions and environments.
Pluralism (creative-productive)	Through interaction diversity arises, and through diversity, unity can arise. Interaction necessitates diversity and that diversity must be recognized.
Community (communal)	Arises from Interaction and Pluralism. Community grounds and limits our realities and ideas.
Growth (enjoyment)	Combines realities and ideas in a constant cycle of change. This change develops communities in positive and negative ways.

Interaction (Martínez: geographical) states that “…organisms such as trees and people are not independent things that occasionally act on others, they are rather constituted by their interactions and so are at once continuous with their environment” (Pratt, 2002:24; Martínez Luna, 2015). This is also reflected in the milpa metaphor that Barkin (2022) uses to illustrate Indigenous comunalidad. All parts of the milpa—an Indigenous agricultural innovation commonly used by Indigenous Americans and known in the US as The Three Sisters—serve to interact and nourish the others. A community is the same, all individual parts work toward benefitting the greater whole and all parts are intricately interconnected through roots (community/network/interactions) that supply the greater whole and the individual simultaneously (Barkin, 2022).

Pluralism (Martínez: creative-productive) adds to interaction by affirming that diversity is as important as unity and must be recognized for the ways that diversity adds to and expands realities. Community (Martínez: communal) follows from the first two dimensions and asks us to remember the “…constitutive role of human communities in knowledge and ontology” (Pratt, 2002:28; Martínez Luna, 2015). In other words, our communities are a grounding and limiting reality for many ideas and realities.

Finally, the dimension of growth (Martínez: enjoyment) combines interactions and realities in constant cycles of change and new understandings, growth moves communities and realities forward and helps to develop them on all levels. Growth does not necessarily mean that all changes or forward momentum are positive and necessarily constructive, growth can also be detrimental to a community; growth is, on a simple level, the process of change (Pratt, 2002:35–36; Martínez Luna, 2015).

Prior to encountering Indigenous pragmatism, the colonial tradition saw the world as literally rising from the stories of the Bible. This strict interpretation of biblical events by colonial scholars and power-players was based on a long history of savagizing Indigenous peoples and viewing them as servants of the devil worthy of domination and oppression (Williams, 2012; Mackay and Feagin, 2022). Biblical stories were further interpreted by colonial scholars to justify the genocide occurring in the Americas as one which was ordained and demanded by the Christian God (Pratt, 2002:37–55) while simultaneously establishing an idea of hierarchical and chronological importance. Early chronological points on the timeline were necessarily primitive, their time had come and gone and are replaced by Euro American society which is allegedly more important and advanced since it arises and continues to occupy future points on this timeline (Pratt, 2002:46–54). This view is in direct opposition to what Pratt calls the “Indigenous attitude” (Pratt, 2002:78) and the Indigenous idea of a cyclical existence and timeline (Rivera Cusicanqui, 2020:48).

Pratt (2002:89–94; 144–162) and Cordova (2007) show that the pragmatic dimensions were present in Indigenous communities, actively taught, and communicated as a way of living peaceably with others different from you and your community for millennia, constituting what Sánchez-Antonio (2022) calls millennia-knowledge. This Indigenous attitude/millennia-knowledge was subsequently communicated to colonial-invaders like Roger Williams and Thomas Morton by Indigenous leaders across the hemisphere. Williams and Morton would later incorporate these ideas into their own Western works and lives to the point that they were actively vilified and aggressively persecuted by other Euro-American colonists for being “race traitors” (Pratt, 2002; Mackay and Feagin, 2022).

Pratt also addresses Euro-American criticisms against Indigenous pragmatism, particularly that the pragmatic sees the world as made up of fractured and unequal, but interacting communities. This criticism reinforces a Western view of the world as necessarily operating in an us vs. them duality—itself an analytic bifurcation or imperial binarism that must be overcome (Gandhi, 2006; Go, 2016). The works of González Casanova (1965), Neolin (Pratt, 2002:154–157), Sagoyewatha (Pratt, 2002:159–162), Tenskwatawa (Pratt, 2002:156–158), Cordova (2007), de Sousa Santos (2014), Martínez Luna (2015), Sánchez-Antonio (2022), and others highlight that such an idea of division and separation is a Western invention fundamentally at odds with the pluralism and acceptance that Indigenous pragmatism calls for in its very structure. This divisional duality inherent in Western thought requires all communities, including Indigenous, to see themselves as set to a standard that can be applied across all people, time, and place (Pratt, 2002:155). This contributes to ongoing internal colonialism that Indigenous communities and knowledge must avoid and overcome (Sánchez-Antonio, 2022).

## Indigenous critique and resistance to the critique

In their wide-ranging book, *The Dawn of Everything: A New History of Humanity* (2021), anthropologist David Graeber and archaeologist David Wengrow, ask us to contemplate how most of human history and civilization has previously been conceptualized.

They argue that Western scholars place far too much focus on the dawn of agriculture and birth of Western-Hellenistic thought as the two generalizable standards of civilization by which all global communities can be judged. Their argument follows from the strong tradition of Global South scholars like Mignolo, Santos, and Sánchez-Antonio who have written extensively on the epistemic oppression of non-Western frameworks—and argued that global social justice can only arise from a global epistemic justice (de Sousa Santos, 2014; Mignolo and Walsh, 2018; Sánchez-Antonio, 2022). Only by seriously engaging with these projects of decolonialism and epistemic justice will Indigenous millennia-knowledge be able to unseat Western knowledge dominance and adequately consider the Indigenous American experience. Graeber and Wengrow call this as an Indigenous critique and believe that it is the path forward. They define it as “…taking seriously contributions to social thought that come from outside the European canon, and in particular from those indigenous peoples whom Western philosophers tend to case either in the role of history's angels or its devils” (Graeber and Wengrow, 2021:5). The Indigenous critique as defined by Graeber and Wengrow is a decolonial turn that centers millennia-knowledge and Indigenous scholars to resurrect Indigenous knowledge (Sánchez-Antonio, 2022).

The late Mexican anthropologist, historian, and United States Library of Congress Living Legend Miguel León-Portilla long led the charge for such a critique. He was widely considered one of the top experts on Mexica culture, literature, and philosophy (Schwaller, 2020:671–677). His book *Aztec Thought and Culture: A Study of the Ancient Nahuatl Mind* (1963)—originally published in Spanish as *La filosof*í*a Náhuatl estudiada en sus fuentes* (1956)—argued strongly for an Indigenous critique. He highlights a 1,524 example where Tlamatinime are recorded speaking to an audience that included Spanish priests, these Indigenous thinkers incisively critiqued the invaders (Friars of Saint Francis, 1564; Léon-Portilla, 1963:63–66):

Perhaps we are to be taken to our ruin, to our destruction. But where are we to go now?

We are ordinary people, we are subject to death and destruction, we are mortals; allow us.

Then to die, let us perish now, since our gods are already dead….and now, are we to destroy the ancient order of life?

…We certainly do not believe; we do not accept your teachings as truth, even though this may offend you… it is not enough that we have already lost, that our way of life has been taken away, has been annihilated. Were we to remain in this place, we could be made prisoners.

Their critique recognizes that the Western framework does not value the pragmatism of Indigenous Americans and instead promotes intolerance. This critique mirrors that of Kandiaronk—I'll discuss him momentarily—and was shared over a century later in the 1640s by the Narragansett leader Miantonomi when he spoke to a gathering of Indigenous people recognizing that, “…we must be one…otherwise we shall be all gone shortly” (Massachusetts Historical Society, 1833:152–155).

Despite ongoing resistance, the Indigenous critique continues to grow across disciplines, however Global North sociology still needs to begin to recognize that such thought has and currently exists, and intentionally work toward incorporating that thought meaningfully. There is no shortage of material from which to develop these ideas. In addition to the critiques of the Tlamatinime we have the critiques of many other Indigenous thinkers and leaders utilizing Indigenous millennia-knowledge. These leaders are listed in Table 2, which is certainly not exhaustive:

**Table 2:** Some Indigenous critics, approximate birth and death dates, and their people/native nation.

**Indigenous critics**		
**Indigenous critic**	**Life**	**People/native nation**
Tlamatinime (Aztec scholars)	Pre and post invasion	Mexica (Aztec)
Nezahualcoyotl	c.1402–1472	Mexica (Aztec) and Acolhua
Miantonomoh	c.1565–1643	Narragansett
Kandiaronk	c.1649–1701	The Wendat at Michilimackinac
Teedyuscung	c.1700–1763	Leni Lenape
Neolin	Uncertain-c.1763	Leni Lenape
Sagoyewatha (Red Jacket)	c.1751–1830	Seneca
Pushmataha	c.1764–1824	Chahta (Choctaw)
Ma-ka-tai-me-she-kia-kiak (Black Hawk)	c.1767–1838	Sauk
Tecumseh	c.1768–1813	Shawnee
Tenskwatawa	c.1775–1836	Shawnee
John Ross	1790–1866	Tsalagi (Cherokee)
William Apess	1798–1839	Pequot
Tocmetone (Sarah Winnemucca)	c.1844–1891	Northern Paiute
Ohíye S'a (Dr. Charles Eastman)	1858–1939	Santee Dakota
Wassaja (Dr. Carlos Montezuma)	c.1866–1923	Yavapai Apache
Zitkála-Šá (Gertrude Simmons Bonnin)	c.1876–1938	Yankton Sioux
Vine Deloria Jr.	1933–2005	Standing Rock Sioux
Viola Faye (VF) Cordova	1937–2002	Jicarilla Apache
Silvia Rivera Cusicanqui	1949-Present	Aymara
Juan Carlos Sánchez-Antonio	1983-Present	Zapotec
Zapatistas	1994-Present	Maya (other Indigenous alliances)

Indigenous oppression has a long and complex history (Stannard, 1993; Williams, 2012; Mackay and Feagin, 2022). It conceptually began with the ancient Greeks (Williams, 2012) and physically began with Christopher Columbus in 1492 and continues today. The genocidal colonialism of what is today called the Americas established a specific view of Indigenous peoples and their cultures, one where they were seen as demonic, savage, and unintelligent (Stannard, 1993; Williams, 2012; Townsend, 2019; Mackay and Feagin, 2022). Denial of Indigenous thought and knowledge still pervades modern academic institutions where the school of thought is still decidedly white, male, and Western (de Sousa Santos, 2014; Mignolo and Walsh, 2018). Even in disciplines such as sociology we see this denial of Indigenous knowledge and communities, after all it took 115 years for the American Sociological Association (ASA) to gain a section on Indigenous Peoples and Native Nations. Sociologists—who should by nature of the discipline be familiar with critiques by oppressed Indigenous thinkers—are well versed in Western scholars and critics such as Marx, Durkheim, Weber, Aristotle, Plato, etc. How many Sociologists would readily attest to at least hearing the names of past and current Indigenous thinkers analyzing race, economics, identity, war, colonialism, philosophy, religion, European society, and more as far back as the beginnings of colonization (Friars of Saint Francis, 1564; Léon-Portilla, 1963:18–21; Graeber and Wengrow, 2021:38–77; Sánchez-Antonio, 2022)? That answer is likely as obscure as Global North recognition of Indigenous thinkers themselves.

The refusal of many scholars to recognize and utilize Indigenous thought advances the deeply engrained stereotype of Indigenous peoples as savages without a valuable intellectual framework or conceptual understanding of the world beyond their primitive and savage existence. This white racially framed narrative—and its well established and explicitly racialized anti-Indigenous subframe (Stannard, 1993; Williams, 2012; Feagin, 2020; Mackay and Feagin, 2022)—of Indigenous knowledge reduces Indigenous thought to savagery and re-interprets it as a product of Western thinkers. This reframing of Indigenous thought as *actually* a product of White Western thought essentially self-Indigenizes any product of Indigenous thinkers as a product of Euro-Whites (González Casanova, 1965), much how 21st century whites are claiming their own distorted indigeneity to preserve colonial and white supremacist policies—the intended results are the same: to erase Indigenous peoples and their knowledge (Leroux, 2019).

## Discussion

Other disciplines have been and still grapple with how to use Indigenous thought and critique as a structure itself, rather than something that takes a secondary role next to the dominant settler colonial knowledge base. The Global North tradition must begin to recognize and familiarize itself with the thought and critique of historical and contemporary Global South scholars, Indigenous and non-Indigenous, who have been doing this work for centuries and were the first to do so in many cases.

Global North sociology must begin to recognize and utilize Indigenous knowledge on an equal footing, while also considering that Indigenous knowledge does not view our reality in the same way. Indigenous scholar Silvia Rivera Cusicanqui illustrates this when she critiques the Western/Global North constructions and obsessions with prehistory, post-colonialism, etc. She says of Indigenous worldviews (2020:48), “There is no *post* or *pre* in this vision of history…rather [it] moves in cycles and spirals and sets out on a course without neglecting to return to the same point. The indigenous world does not conceive of history as linear; the past-future is contained in the present”.

This rejection of a linear timeline by Indigenous thought can be seen in the struggles of Indigenous groups and regions like the Zapatistas, American Indian Movement, Chéran, and others that are currently attempting to rebuild Indigenous thought and society. These communities see the future of Indigenous communities and millennia-knowledge as necessarily involving resurrecting language, thought, culture, and more to truly save Indigenous peoples from the ongoing onslaught of colonialism (Sánchez-Antonio, 2022). This epistemic justice (de Sousa Santos, 2014) is vitally important for Indigenous millennia-knowledge to overcome dominating Western thought and paradigms. Indigenous scholars and movements can fall prey to internal colonialism that reifies the very colonial structures it attempts to fight against by not adequately resurrecting and implementing Indigenous millennia-knowledge (González Casanova, 1965; Sánchez-Antonio, 2022). Indigenous thought must constitute the very foundation of Indigenous struggle and movements, inside and out of academia, otherwise it is based on colonial and postcolonial ideologies that merely reinforce the very elements that the struggle is attempting to overcome.

Using these Indigenous concepts on an equal footing is grounded in another pluralist Indigenous philosophy that comes from the Mi'kmaq. Etuaptmumk—translated as two-eyed seeing (Kutz and Tomaselli, 2019). It recognizes that knowledge is cogenerated, and we learn to see “from one eye with the strengths of Indigenous knowledges…and from the other eye with the strengths of Western knowledges…and [using] both these eyes together, for the benefit of all” (Marshall, 2004).

## Systemic racism and multi-raciality

We can ponder, how would Indigenous pragmatism interact with the theory of systemic racism (Feagin, 2006)? The dimensions of interaction, pluralism, community, and growth were applied by Indigenous thinkers to critique and analyze a systemically racist system that they could see was imported and evolving with the explicit intent of complete Indigenous elimination. Colonial academic violence and erasure continues to employ white racially framed narratives that Indigenous peoples are largely passive bodies and actors in the process of colonialism, an assumption that obfuscates the determined Indigenous resistance to erasure. The Indigenous critique is not merely an early and contemporary recognition of violent Euro-American actions, but an explicit recognition of a systemically racist society that “others” Indigenous people to such an extent that their violent elimination is justified (Mackay and Feagin, 2022). This recognition of the organized and institutionalized racism and anti-Indigenous subframe were explicitly relayed in public criticisms of Euro-American society and its frameworks by Indigenous groups like the Tlamatinime, leaders like Miantonomi, and many others (Friars of Saint Francis, 1564; Pratt, 2002).

Systemic racism and its empirical puzzles can be analyzed using the millennia old Indigenous pragmatic. The dimensions of Indigenous pragmatism (see Table 1) would say that we must accept diversity in our institutions and society. This should be a necessary condition of their very existence. The Indigenous pragmatic demands that dismantling racist systems entails utilizing the Indigenous pragmatic dimensions of interaction, pluralism (unity through diversity), community, and growth (learning from one another on an equal plane). Through this plurisociety it is possible to develop a community that is based on the very intention of non-discrimination and non-oppression. What this would look like in reality would likely be difficult to predict, largely because Indigenous millennia-knowledge has not been widely implemented and is in a constant state of offense-defense against colonial attack, however communities in Chiapas (Zapatistas) and Michoacán (Chéran) have shown that governments and communities based on Indigenous thought and structure are possible, peaceful, and can thrive for all that live there (Barkin, 2022).

For a different example, let us turn to racism in medicine which is a pervasive problem (Feagin and Bennefield, 2014) and has been particularly relevant to Indigenous communities even before the current COVID-19 pandemic. US Indigenous communities, particularly on reservations, rely on the federally funded and run Indian Health Service to provide for their well-being due to treaty obligations agreed to by Indigenous governments and the US. However, the IHS has failed in many respects and remains a systemically racist and sexist institution (US Commission on Civil Rights, 2004; Gurr, 2014) run by a settler colonial government that frequently ignores treaty obligations (Echo-Hawk, 2013). How would the Indigenous pragmatic provide a framework for evaluating issues of systemic racism in medicine?

Through the principle of interaction, it is possible to understand that the interactions of Indigenous peoples working in and visiting the IHS facilities are providing important and crucial social interactions with non-Indigenous employees and IHS officials that can build community. These provider—patient interactions are an important part of medicine, however the problem of providers in the IHS providing culturally irrelevant care contributes to the systemically racist structures within medicine overall and by extension, the IHS (Feagin and Bennefield, 2014; Mackay, 2022). Recognizing and implementing the dimension of interaction also must incorporate the Indigenous relations to land, nature, and community that are components of effectively treating the overall patient, a concept now popularly termed One Health (United States Centers for Disease Control Prevention, 2022; Mackay, 2022).

These interactions lead to pluralism which recognizes that diversity necessarily exists in a world that has multiple lived experiences (realities) and multiple ways of relating to one another and land. Medicine may superficially recognize interactions and their importance, but pluralism can help medicine accept and implement the necessity for diversity and thereby incorporate a level of unity into medical systems, particularly one like the IHS which has a specific mandate to treat Indigenous communities. Through interactions and pluralism there can develop the dimension of community. As medical systems currently stand there is a level of removal from the patient. The Western system generally does not effectively integrate comunalidad, in turn this means that medical systems treating Indigenous people are not grounded in the lived realities whereby Indigenous people value community responsibility and involvement in their medical care (Mackay, 2022). Further, the Indigenous pragmatic dimension of growth comes from the previous three dimensions. Since medical systems do not effectively integrate the Indigenous pragmatic, that same pragmatic would argue that it is fundamentally impossible to create positive growth within these medical systems as they currently stand. Future research that considers the pragmatic and its dimensions can address an argument that current medical systems are nonsensical under this worldview and could provide for unique and innovative solutions and medical systems.

Extensive research in sociology has shown that the US is a systemically racist and settler colonial society, but that research has not analyzed these systems through the lens of Indigenous philosophy whereby the racist systems themselves are fundamentally flawed and illogical. The Indigenous pragmatic as a mode of analysis can help future research to argue that these systems, based on philosophies, must be overturned as they are fundamentally nonsensical within Indigenous philosophies. While I have used systemic racism in medicine as a specific example here, the pragmatic can be extended to many various empirical puzzles related to systemic racism, for example: systemic racism and sports, systemic racism and women's rights—itself an important component of Indigenous liberation and sovereignty (Lugones, 2008; Sánchez-Antonio, 2022), systemic racism and rights of LGBTQ populations, systemic racism and debates of settler identity, and more.

## Multi-raciality

Another potential analysis—interacting with systemic racism and arising from an Indigenous framework that utilizes Indigenous pragmatism and ch'ixi—could be how multiracial individuals living in colonial societies, such as myself, view and mediate our racialized identities. Rivera Cusicanqui (2020:51–60), herself mixed-Indigenous, details how multi-raciality and multi-culturalism without ch'ixi does not ultimately do enough to recognize that all parts of an identity are equal and carry equal weight in order to create a new and unique individual. The critique that current literature does not do enough to analyze multi-racial experiences and negatively racialized individuals—and new terms and frameworks are needed—was recently recognized in a scholarly paper on multi-raciality. The authors state, “…new theoretical frameworks specific to Multiracial families are necessary…” (Atkin and Yoo, 2019). Ch'ixi can help fill this recognized gap in the literature and advance the field as it is a specific framework that centers the concept of multi-raciality, its positive and non-racialized aspects, and associated lived experiences. It may additionally help multi-racial individuals to accept their multi-racial ancestry and more fully embrace their rich and diverse heritage when they otherwise may be hesitant, hostile to that ancestry, or employ methods such as code-switching in a society that negatively racializes multi-raciality (Anzaldua, 2012; Gonlin, 2022).

Anzaldua (2012) highlights code-switching in her analysis of borderlands and the concept of nepantla (Nahuatl: in the middle of it; between) to describe in-betweenness of lived experiences and identities, particularly in border communities. Chi'ixi would likely disrupt this idea of nepantla as it has currently been conceptualized in the literature. Nepantla—based on its currently associated code-switching dimension—demands switching between identities, essentially a duality of Indigenous vs. non-Indigenous. Under Ch'ixi someone is all their identities at once, the switching of identities—or the Western demand of “picking” one identity for mixed people—is illogical and fundamentally unnecessary under Ch'ixi. Utilizing this understanding of identity is one way that mixed individuals can understand and embrace their identity, however it must also be used in conversation with the overall systemically racist structure in which it exists. Code-switching dualities can be used in a systemically racist system that does not encourage the Indigenous pragmatic and acceptance of unity through diversity. Sometimes it can be necessary to employ code-switching for an individual's safety. The impact of integrating an Indigenous pragmatic and Ch'ixi in a systemically racist society could alter how mixed individuals interact with their communities and how communities interact with them. Complications such as this are what make Indigenous knowledge and frameworks so necessary and exciting to explore, Ch'ixi provides a potentially liberating avenue.

Relatedly, Rivera Cusicanqui is quite critical of the concept of multi-culturalism, especially as it relates to Indigenous peoples. She insists that the current constructions of multi-culturalism and multi-raciality-without-Ch'ixi are (2020:56–57):

…Essentialist and historicist interpretations of the indigenous question. They do not address the fundamental issues of decolonization but instead obscure and renew the effective practices of colonization. Their function is to supplant the indigenous populations as historical subjects and to turn their struggles and demands into elements of a cultural reengineering and a state apparatus in order to subjugate them and neutralize their will.

Rivera Cusicanqui's firm critique of multi-culturalism and multi-raciality-without-Ch'ixi recognizes that Indigenous peoples and identities are still actively racialized, ignored, and removed from these conversations—something that other Indigenous scholars have recognized as well (Oviedo-Freire, 2021; Sánchez-Antonio, 2022). Rivera Cusicanqui recognizes that any conversation or framework—even well intentioned multicultural ones—that includes Indigenous peoples does so through a colonial lens and does so in order to continually subjugate Indigenous people, their identities, and to hold them to a universal colonial standard, a violation of the Indigenous pragmatic (Pratt, 2002) and a viewpoint that can likely court passionate discussion among scholars in future research.

However, as Rivera Cusicanqui states, these ideas have largely been crafted by non-Indigenous American scholars and implemented within colonized spaces. This means that Indigenous peoples are excluded, and Indigenous identities are subjugated under others. In a colonial framework that does not incorporate Indigenous concepts of identity, the Indigenous identities of individuals and communities are automatically and necessarily oppressed. To effectively liberate Indigenous people a concept and system must be implemented that elevates Indigenous identity by its very existence and core conception. Under Indigenous millennia-knowledge that elevation is possible as its philosophy sees multi-racial Indigenous people, and other mixed individuals, as positive and something beautifully unique—an included third that combines all aspects of our realities and communities into an identity/individual that can interact with and grow all communities and realities. Again, Zapatista communities, Zapotec/mixe movements, and the town of Chéran all show that this Indigenous approach is necessary for Indigenous liberation and decolonial actions to effectively work (Barkin, 2022; Sánchez-Antonio, 2022; Schools for Chiapas, 2022).

These areas of research will be critical as the US multiracial population continues to grow and society must increasingly incorporate Ch'ixi identities. As this research continues to gain steam and attention, the risk is that the contributions of Indigenous thinkers and ideas regarding multi-raciality/culturalism will be internally colonized and regurgitated as a new Euro-American idea to analyze the demographic shifts in the United States (González Casanova, 1965). Early and continuous recognition that Indigenous scholars and knowledge, as well as other Global South scholars, have created these ideas and initiated these conversations will be extremely important.

## Postcolonial thought

Indigenous frameworks can add depth and nuance to movements such as the postcolonial theoretical movement. Julian Go's 2016 book on the topic provides an important introduction to postcolonial thought while highlighting its pluses and minuses and the major figures within the subaltern movement. However, even the postcolonial movement maintains a focus everywhere but on the Indigenous peoples of the Americas. Indigenous American thought is barely mentioned, and Indigenous American scholars are equally rarely mentioned. Indigenous scholars like Jairo Fúnez-Flores have highlighted that postcolonialism still maintains a largely Eurocentric/Francocentric focus (Fúnez-Flores, 2022) conceptually and in practice. This is important because Indigenous pragmatism and thought are present in the very ideas that Go mentions as core to the postcolonial framework, even unintentional omissions such as this continue to perpetuate internal colonialism and their omission weakens analyses of Indigenous communities under these frameworks. Pablo González Casanova addresses this when he states that Indigenous peoples and scholars must “…consume, in a regurgitated form, the very ideas…that we indigenous people and intellectuals…have produced independently” (González Casanova, 1965; Rivera Cusicanqui, 2020:61).

In a section titled “Turning South, Going Native” (Go, 2016:147) the author states, “Simply put, indigenous sociology aims to globalize social science by mining currents of thought from outside the metropole and using them to reorient social theory”. Despite the racialized and co-naturalized racio-linguistic history of phrases like *going native* (Rosa and Flores, 2017)—and the colonialist metaphor and history of mining Indigenous resources—Go perfunctorily highlights the importance of using Indigenous knowledge as knowledge in and of itself. He calls for the use of perspectival realism, a Western based philosophical perspective that demands that “truths of knowledge are always partial, and…depends on the observer's position” (Go, 2016:163) while assuming that “…no theory is universal” (Go, 2016:182). This call for application of perspectival realism does not mention how it impacts and interacts with the ideas of Indigenous American philosophies. Go (2016:143) cites Leela Gandhi and Edward Said in arguing that we must overcome the binaries and dualisms we have become used to in imperial settings, yet this binarism still exists in that Go fails to recognize or analyze in any meaningful way how Indigenous American thought may have contributed to this project.

For example, Go states that perspectival realism finds that truths are always partial and come from the perspective of the individual or community observing. Indigenous frameworks would agree with this, but Indigenous frameworks would also argue that *truth* is inherently and necessarily unknowable in its entirety and we cannot know the full truth as we are existing in a world that does not always need our classification and pursuit of a dominant truth. The partial truths of perspectival realism under an Indigenous framework are not invalid or damaged in their assertions, rather they are an addition to a rich tapestry that must include all partial truths, communities, and realities. We should celebrate the partial truths and accept their unique validity rather than view them as an obstacle to overcome or otherwise “deal with.” However, Indigenous philosophy would also argue that through Etuaptmumk it would be possible, even if difficult, to incorporate those partialities to see a larger picture. While perspectival realism seems to imply that there is always a corrupting partiality in everything, Indigenous philosophy embraces that partiality and requires it to see the larger picture with both eyes.

Additionally, the relatively modern perspectival realism states that no theory or truth can be seen as universal, that everyone perceives things differently. However, this method still stems from Western conceptions that each person views reality from a common standard (e.g., the biblical Golden Rule explored in the Religion section below). Indigenous philosophy rejects this universal basic standard, this means that we cannot judge societies and beings along a singular and linear timeline or to a singular standard, an idea already present in Indigenous millennia-knowledge. As Pratt (2002) and Sánchez-Antonio (2022) show, Indigenous philosophy necessarily allows for a basic pluralism that is rarely present, or actively suppressed, in Euro-Western thinking. This pluralism celebrates unity through the diversity of thought and tradition and regularly supports that multiple truths, theories, basic standards etc. can peaceably co-exist and must exist to adequately consider reality. This non-universal realism of thought and community can lead to growth that postcolonialism calls for in sociology but from a common standard, Indigenous pragmatism rejects this common standard and views partiality/non-universalism not as an abstract theory or obstacle to explanation.

The question then remains: can postcolonial thought be effectively utilized for liberation of Indigenous American communities and thought? Rivera Cusicanqui (2020:48) makes her thoughts clear on the matter. She argues that the “post” in postcolonial implies a linear and hierarchical ordering of time and society, Indigenous worldviews across communities reject this idea of a universal linear timeline, therefore a postcolonial approach makes no logical sense for Indigenous thought. Indigenous scholars are instead calling for decolonial approaches as this recognizes an Indigenous worldview that past, present, and future exist and influence one another simultaneously and that actively rejects any aspect of colonial domination over Indigenous communities and thought (Mignolo and Walsh, 2018; Rivera Cusicanqui, 2020; Sánchez-Antonio, 2022). Analysis of systemically racist and anti-Indigenous structures in the US are not effective unless they recognize that we are not living in a *post*-colonial society, this makes the present colonial structures and the associated systemic racism opaque. If we are to analyze Indigenous American issues under a postcolonial framework, then we are subsuming Indigenous communities under yet another colonial framework that ultimately denies Indigenous liberation. If we momentarily return to systemic racism in medicine, we can illuminate some of this trouble.

Postcolonialism, by its very definition, would see the IHS as a relic of previously colonial policies and institutions in a binarism of pre vs. post. This negates the still valid treaties around the IHS between the US and sovereign Indigenous governments as well as diminishing the impact that the settler-colonial US government has on the IHS and Indigenous health. The Indigenous pragmatic and an Indigenous conception of time necessarily sees the IHS not as a postcolonial institution and system, but rather as a system that is made up of its colonial past, present, and future. Therefore, analyses of the IHS under postcolonial approaches cannot adequately account for the larger picture in which the IHS exists. Systemic racism in medicine analyses can use Indigenous millennia-knowledge to effectively analyze Indigenous and marginalized experiences in colonial medical systems more appropriately and account for the particulars of Indigenous experiences within Western medicine (Mackay, 2022).

This same idea applies to settler-societies at large. The place of Indigenous people within nations like the US, Canada, Australia, Mexico, and Brazil is one of subjugated and oppressed marginality. These countries are still dominated by and based on settler-colonial institutions that have not and cannot enter a post phase, they are still very much alive and well. Indigenous truth would not demand that sociologists discover every granular detail of the Indigenous experience in these societies; rather Indigenous truth and the Indigenous pragmatic would argue analysis of systemically racist structures across society must not conceptualize Indigenous oppression and history as relegated to a post era or attempt to fit Indigenous experience and oppression completely into frameworks developed in and by those same colonial institutions. The present and future are very much influenced by colonialism and newly created structures and analyses developed from these ideas and institutions are still perpetuating ongoing colonialism in new forms.

## Religion

Pratt (2002) highlights how the Indigenous pragmatic also incorporates what he calls a logic of land. This logic is discussed throughout his book, but one Indigenous criticism is quite pertinent as it reveals the Indigenous pragmatic at odds with core Christian ideas, particularly the Golden Rule. Pratt introduces readers to interactions of the Delaware leader Teedyuscung with a Quaker missionary. The missionary explains to Teedyuscung that the rule is “for one man to do to another as he would the other should do to him” (Pratt, 2002:163). Teedyuscung considered this and after a short time came back to the missionary and explained that this rule was impossible since creationary forces would need to instill humans with “a new heart” (Pratt, 2002:163). This doesn't imply that Teedyuscung considers the world too selfish to apply the rule, but rather that the rule itself is inadequate and constraining. The Golden Rule implies that the two people are coming from identical visions of fairness and reality, however Indigenous thought recognizes that this is almost never the case and should never be assumed, plurality must be accepted and assumed instead. Teedyuscung is arguing that the two people should be using two-eyed seeing instead, through seeing where both individuals are coming from it would produce a result that is more equitable and agreeable to both parties. The Golden Rule as it stands is essentially one-eyed seeing.

Teedyuscung also uses the Indigenous pragmatic in a more nuanced way to criticize the larger colonial objectives and Euro-Western demands that subsume all people under a singular method, assume that identical standards should and must be applied to all people, and see the land as merely a resource to exploit. By demanding that all people are of the same ethic and development the Golden Rule removes the logic and importance of the Indigenous pragmatic—and a logic/interconnectedness to land—to Indigenous communities. This further reduces land, and climate (Barkin, 2022), to the resource and value extraction of Euro-Western thought and provides a base to justify violent Indigenous dispossession (Pratt, 2002:166).

Just as the Tlamatinime recognized how Christianity would affect Indigenous lives and culture (Friars of Saint Francis, 1564; Léon-Portilla, 1963:63–66), Teedyuscung also recognized contradictions between Christianity and Indigenous thought. Research into society and religion, particularly in the Americas, must interact with colonialism and what that means to Indigenous people, the idea that Indigenous people did not resist colonialism or viewed Europeans as gods is false and a colonial/Christian fiction invented by the invaders themselves (Restall, 2004:97–98; Townsend, 2019:95–111). Sociological analyses of religion can be buoyed by taking a specifically Indigenous approach. Future research could do more than recognize that Indigenous people resisted, it could also include the Indigenous criticism of Christianity based on the Indigenous pragmatic, just as Teedyuscung does with his critique of Christian principles. This is not to say that future research must say that Indigenous people rejected Christianity, though many did, rather that Indigenous peoples saw the contradictions within Christian thought and action and criticized it based on their own traditions, histories, and knowledge. Under the Indigenous pragmatic this was not an issue, Christianity, just as any idea, could be criticized, debated, and considered.

This tradition of healthy debate concerning all matters, including Christianity and its precepts, was exemplified through Kandiaronk who famously irritated Jesuit priests and European intellectuals with his intelligence and wit (Graeber and Wengrow, 2021:48–56). Kandiaronk frequently criticized Christianity and engaged in debate with missionaries around him. He recognizes the disconnect between Euro-Christians and Indigenous traditions saying, “It's only natural for Christians to have faith in the holy scriptures, since, from their infancy, they've heard so much of them. Still, it is nothing if not reasonable for those born without such prejudice, such as the Wendats, to examine matters more closely” (Lahontan, 1703; Graeber and Wengrow, 2021:52). Interesting future research could analyze Kandiaronk's varied criticisms of Christianity and European culture such as his points: one religion for all people doesn't make sense (Graeber and Wengrow, 2021:53); Eternal damnation makes no sense as humans are not inherently bad, but rather a religion and culture that encourages selfish and acquisitive behavior is (Graeber and Wengrow, 2021:53); that money, property rights, and material self-interest leads to an inhuman society such as that of the Europeans (Graeber and Wengrow, 2021:54); and the complete dismantling of European social systems is the only way to solve their many problems (Graeber and Wengrow, 2021:54).

Kandiaronk's critique appears to violate the plurality and two-eyed seeing of Indigenous philosophy. However, Kandiaronk has viewed his culture and European culture through two-eyed seeing and can see that Europeans are unlikely to extend such an open-minded courtesy to Indigenous people. He indicates this when he states that Indigenous people use two-eyed seeing to analyze the benefits and drawbacks of Christianity, however this same vision is not present among Europeans who see Christianity and European culture as infallible and unquestionable. He also reveals this rigid structure in his comments on European society needing a complete reset to better understand and care for themselves and others. The influence of the Indigenous pragmatic relayed to Lahontan by Kandiaronk even extended to the theorizing of French philosopher Jean Jacques Rousseau who plagiarized these Indigenous ideas in his influential works on democracy, freedom, and ethics (Launay, 2018). Rousseau's work was massively influential in European and North American high society and became foundational for many of the “democratic” ideas that influenced the founders of the US. However, this Indigenous contribution, and many others (Mackay and Feagin, 2022), to European democratic thought is rarely if ever recognized, another example of internal colonialism whereby Indigenous peoples experience their own ideas and theories as if they are not their own (González Casanova, 1965). As a political, military, and intellectual leader Kandiaronk saw that he needed to pivot his strategy to one where he actively refutes European society and methods in a desperate attempt to prevent the annihilation of Indigenous ways of life (Graeber and Wengrow, 2021:48–60).

A potential path for future research concerning Indigenous people and religion, particularly Christianity, could sociologically consider how the Indigenous conception of creation differs significantly from Western traditions. Every Indigenous community has narratives of how the world and the cosmos came to be, but similarities are shared across stories and these similarities are beautifully showcased in the ancient K'iche' book the Popol Vuh. The Popol Vuh is one of the most important Indigenous books remaining after Western invaders attempted to obliterate Indigenous cultures. The Popol Vuh is a living pre-invasion document that has been continually updated, including throughout invasion. It is difficult to compare the Popol Vuh to Western conceptions of literature, however the most common comparison made—to help non-Indigenous understand the importance and breadth of the book and information within—is to the Bible. Not a perfect comparison, but it relates the importance of the text, nonetheless.

Part One of the Popol Vuh specifically highlights the concept of creation in a way that is in direct opposition to typical Christian conceptions. The Popol Vuh specifically highlights the trial-and-error process of the Gods when they were attempting to create the earth, plants and animals, and humans. Contrasting Christian conceptions of godly perfection, the Popol Vuh celebrates the mistakes, trial, and error of the Gods. The Indigenous conception of creation here—which interestingly is like many other Indigenous creation stories of divine trial and error—is explicitly scientific. This opposes Christian conceptions of creation and Christianity's ongoing relationship to science, the Indigenous worldview doesn't view the Gods as perfect beings, rather they are individuals that can and do make mistakes and errors. The Popol Vuh explicitly states that the approach of the Gods was neither perfect nor arising out of divine perfection, unlike the Christian God who is viewed as infallible. The Popol Vuh says of creation and the Gods (Tedlock, 1996:64–66):

They are great knowers, great thinkers in their very being….

By their genius alone, by their cutting edge alone they carried out the conception….

Such was their plan when they thought, when they worried about the completion of their work….

In their quest to create beings that could speak and honor the Gods, the animals were created. The Gods recognized their errors and say: “It hasn't turned out well…since it hasn't turned out well and you haven't spoken, we have changed our word…” (Tedlock, 1996:67). The Gods tried again (Tedlock, 1996:68–69).

And then they wanted to test their timing again, they wanted to experiment again, and they wanted to prepare for the keeping of days again….

Again there comes an experiment with the human work, the human design…so then they dismantled, again they brought down their work and design.

This conception of the Gods as beings that can, do, and even should make mistakes indicates a certain worldview whereby the Gods are not infallible beings that know all and create things perfectly. Humans are not explicitly made in their image; in fact, the Gods experiment with multiple different forms of humans. This scientific approach to the story of creation could provide for future research that analyzes the Indigenous relation to Western science. Indigenous peoples have suffered greatly at the hands of Western science and still battle fiercely with Western scientific institutions that inappropriately use Indigenous data or desecrate Indigenous bodies. Modern Indigenous organizations like the Native BioData Consortium are run by Indigenous scientists and community members to ensure that Indigenous data is used appropriately. However, the systemically racist issues surrounding Western bioscience and its exploitation of Indigenous people has created much reluctance by Indigenous peoples to work with scientific groups. This reaction is easily weaponized by colonial systems to claim that Indigenous peoples don't appreciate or understand science, however this is fundamentally untrue. The very creation stories of Indigenous peoples are the definition of science and the scientific method: hypothesis, design, test, observe, report. In these Indigenous creation stories of the experimentation of Gods there is an inherent appreciation of science, one that Christianity actively eschewed and continues to aggressively rebuff.

Analyses of Indigenous peoples, science, medicine, and racism can further consider how this fundamentally scientific belief of Indigenous peoples influences their relation to science overall and scientific concerns like climate and environmentalism (Barkin, 2022). The Indigenous appreciation for experimentation combined with philosophies like the Indigenous pragmatic that values knowledge creation and diversity seem to indicate that Indigenous relations with Western science are far more nuanced than has been considered. Future research into the sociology of science can explore this fascinating connection and potentially provide valuable insights for alleviating the systemic racism present in Western science. This connection could also aid ongoing efforts to encourage young Indigenous people to enter careers in STEM fields by highlighting how their cultures have long held immense appreciation for science and engineering. Here is also an important opportunity to use Etuaptmumk by incorporating the Indigenous appreciation for science—as a fundamental fact of life and creation—with a Western scientific system; this interaction could produce science that is less colonial and racialized. This would allow for two-eyed seeing of empirical puzzles, one from the Indigenous point of view and the other from a Western point of view, thereby increasing the potential for innovation and imagination (Barkin, 2022).

In addition, studies of religion within sociology may want to engage with the Indigenous pragmatic and Gordon Allport's concept of intrinsic and extrinsic religious attitudes and value frameworks (Allport and Michael Ross, 1967). Building on Allport's previous research, Allport and Michael Ross show in their 1967 paper—supported by regional data from research on southern Baptists (Feagin, 1964)—that a person with “intrinsic” religious attitudes views religion more as a lifestyle compared to a person with “extrinsic” attitudes who views religion as more of a networking or business opportunity (Allport and Michael Ross, 1967:434–435). Interesting results from this research (Feagin, 1964; Allport and Michael Ross, 1967) show that those who have an intrinsic religious value framework are less likely to admit to prejudiced attitudes compared to the extrinsic religious individual. The connection here can be made to the long history of religion (e.g., Doctrine of Discovery; Inter Caetera; Spanish missions; English missions; French missions; Manifest Destiny; etc.) exploiting and explicitly advocating for the oppression of Indigenous peoples (Mackay and Feagin, 2022). The Doctrine of Discovery, based on the Inter Caetera (Pope Alexander VI, 1493), states that Indigenous people have no inherent right to their lands, unlike Europeans who allegedly have a holy right to all land across the world, an idea that was reified by the United States Supreme Court—*Johnson's Lessee v. McIntosh*, 21 U.S. 8 Wheat.543 (1823). The Doctrine of Discovery's predatory ethic of Indigenous elimination and dispossession (Williams, 2012; Mackay and Feagin, 2022) directly impacts the US' obsession with Manifest Destiny and western expansion beyond the Mississippi. A destiny that directly led to horrors like the Trail of Tears and many other genocidal atrocities committed by the US government and settler-invaders (Stannard, 1993).

How does the concept of someone living their religion (i.e., intrinsic religious framework)—and therefore allegedly less prejudiced—complicate and interact with the history of colonization and religion? Future research could reengage with Allport's ideas and potentially investigate if this still holds steady under colonial structures. This now decades old research showing that intrinsic-religious and regular church attendees are allegedly less prejudiced (Feagin, 1964; Allport and Michael Ross, 1967) could be complicated by ongoing settler-colonialism. The almost-zealot church attendees and leaders in colonial times who advocated for Indigenous elimination (Mackay and Feagin, 2022) do not seem to fit into this structure. Rather a certain predatory ethic seems to have been at play, despite their regular church attendance and “living” their religion they seem to have harbored extreme prejudice rather than displaying less prejudice, as Allport theorized (Allport and Michael Ross, 1967).

This is not a “post” problem either, this anti-Indigenous zealotry by regular church attendees still impacts Indigenous communities. Recently, a Christian missionary within the borders of the Oglala Sioux distributed a pamphlet denouncing Indigenous culture and beliefs as false and demonic (Associated Press News, 2022). This is but one of many incidents across the centuries that show the value and need for researching anti-Indigenous framing by religious groups, in particular Christianity, from the Indigenous pragmatic—which would advocate for religious plurality.

Indigenous scholars and leaders like Kandiaronk utilized the Indigenous pragmatic and Indigenous knowledge to critique this ongoing religio-prejudice. Kandiaronk's critique of the Euro-Western Christian predatory ethic is essentially an early critique of Allport's ideas centuries later. It would not be unreasonable to think that Kandiaronk would view recent events, and the idea that regular church attendees are less prejudiced, as at odds with what he was experiencing, as he also experienced the prejudice and aggressive reactions of the Christians—clergy and non-clergy alike—to him, his culture, and his people.

## Conclusion

The idea of an Indigenous critique is only recently becoming recognized by non-Indigenous and Global North scholars as a legitimate interrogation of colonialism, society, and more, but it has been crafted since the earliest days of invasion. The critique is based on established Indigenous philosophies that influenced the lives and societies of tens of millions of Indigenous people before European invasion. Previous research by Western/Global North scholars has not adequately incorporated this millennia-knowledge as a structure of its own, but rather as more of a historical curiosity. This paper was never meant to be an answer to all questions, nor to be the final word on Indigenous knowledge, or to speak for all Indigenous peoples. Such assumptions are in direct violation of the very basic foundations of Indigenous philosophy. What I can offer is a way of seeing the clouded reality we exist in: how I see the potential interactions of sociological Indigenous thought, the pluralism of this paper and other ideas, the communities that can benefit from an introduction to these ideas, and finally the growth that I hope will come out of this introduction.

An Indigenous critique, millennia-knowledge, and pragmatic should—and must—be of interest to a broad sociological and scientific audience. The Indigenous critique incorporates Indigenous philosophies and social critiques of many Indigenous people, scholars, and leaders past and present. As the sociology of the Global North begins to reckon with its ongoing erasure of Indigenous people and knowledge, the Indigenous critique can be a liberating way for Indigenous peoples and scholarship to break from the bonds of Western thought and interrogate questions, processes, institutions, and more from an Indigenous perspective, history, and critical lens. Through resurrecting and constructing knowledge systems that are based *on* Indigenous knowledge rather than *of* Indigenous knowledge, scholars can begin to more fully interrogate the experiences of Indigenous communities and potentially find new questions and discussions. Constructing and recognizing Indigenous critiques does not mean that other theories and methods are abandoned, simply that we must see with two-eyes and demand that Indigenous millennia-knowledge be used in conversation with other concepts like systemic racism, identity studies, and postcolonial theory. Indigenous scholars and knowledge must be carefully consulted and utilized so as not to be co-opted or subsumed by the internal colonialism of a nation, but the beauty of the Indigenous pragmatic is that it allows for the pluralism, Ch'ixi, and Etuaptmumk needed to bring Indigenous knowledge onto an equal level.

## Data availability statement

The original contributions presented in the study are included in the article/supplementary material, further inquiries can be directed to the corresponding author.

## Author contributions

RM is the sole author and completed all work on this project. RM retrieved documents, articles, and books and provided the analysis. He found the intertwining and recurrent themes and applied them in the discussion. Author contributed to the article and approved the submitted version.

## Conflict of interest

The author declares that the research was conducted in the absence of any commercial or financial relationships that could be construed as a potential conflict of interest.

## Publisher's note

All claims expressed in this article are solely those of the authors and do not necessarily represent those of their affiliated organizations, or those of the publisher, the editors and the reviewers. Any product that may be evaluated in this article, or claim that may be made by its manufacturer, is not guaranteed or endorsed by the publisher.
